# Risk factors for mortality and severe morbidity in fetuses with normal late third‐trimester scan: population‐based cohort study

**DOI:** 10.1002/uog.29256

**Published:** 2025-06-16

**Authors:** E. D'Alberti, S. Dockree, M. Garbagnati, C. Granieri, A. Cavallaro, L. Impey

**Affiliations:** ^1^ Women's Centre, John Radcliffe Hospital Oxford University Hospitals NHS Foundation Trust Oxford UK; ^2^ Nuffield Department of Women's & Reproductive Health University of Oxford, University of Oxford Oxford UK; ^3^ Department of Maternal and Child Health and Urological Sciences Sapienza University of Rome Rome Italy; ^4^ Department of Obstetrics and Gynecology Università Cattolica del Sacro Cuore, Fondazione Policlinico Universitario A. Gemelli IRCCS Rome Italy

**Keywords:** adverse perinatal outcome, antepartum surveillance, stillbirth, universal scan

## Abstract

**Objective:**

To identify antepartum risk factors for fetal or neonatal mortality and severe morbidity in pregnancies undergoing a universal late third‐trimester ultrasound scan in which there was no ultrasound evidence of small‐for‐gestational age (SGA) or fetal growth restriction (FGR).

**Methods:**

This was a retrospective population‐based cohort study of singleton, non‐anomalous term pregnancies undergoing a universal ultrasound scan at 35 + 1 to 36 + 6 weeks' gestation at a major UK maternity unit, over a period of 7 years. Pregnancies complicated by SGA or FGR were excluded. The outcomes were: stillbirth; severe composite adverse outcome, including extended perinatal mortality and severe morbidity; and severe SGA, defined as birth weight < 3^rd^ centile. Potential clinical, demographic and ultrasonographic risk factors were evaluated using univariate and multivariate logistic regression analysis.

**Results:**

The study population of 40 169 pregnancies comprised 88.9% of all eligible term pregnancies. There were 48 (0.1%) stillbirths, 221 (0.6%) cases of severe composite adverse outcome and 295 (0.7%) cases of severe SGA. Five (10.4%) stillborn infants weighed < 10^th^ centile at delivery (adjusted odds ratio (aOR), 2.70 (95% CI, 1.06–6.92)); 23 (47.9%) cases of stillbirth were considered unexplained. Pre‐eclampsia was associated with stillbirth (odds ratio, 4.46 (95% CI, 1.99–9.96)) but no other antenatal factors, including ultrasound measures of fetal growth, showed a significant association with this outcome. Pre‐eclampsia constituted 9.6% of the population‐attributable fraction for stillbirth. Severe composite adverse outcome was associated only with pre‐existing diabetes (aOR, 2.82 (95% CI, 1.02–7.85)) and nulliparity (aOR, 1.56 (95% CI, 1.19–2.04)).

**Conclusions:**

The potential to predict mortality and severe morbidity in fetuses without ultrasound evidence of SGA and FGR is poor. In these fetuses, the role of ultrasound in further reducing adverse outcomes at term is limited. © 2025 The Author(s). *Ultrasound in Obstetrics & Gynecology* published by John Wiley & Sons Ltd on behalf of International Society of Ultrasound in Obstetrics and Gynecology.

## INTRODUCTION

The prevention of stillbirth relies heavily on ending a pregnancy by medically‐indicated induction of labor or Cesarean delivery after identification of elevated risk. In the preterm period, the risks associated with these interventions are considerable, so the threshold for intervention is high; at later gestations, the risks are lower and intervention is common. Nevertheless, even term birth before 39 weeks' gestation is associated with adverse neonatal and childhood outcomes^1^. As most pregnancies reach term, the impact of increased frequency of early term birth could be substantial at a population level.

Most stillbirths in early pregnancy are of small‐for‐gestational‐age (SGA) fetuses but, at term, the prevalence of SGA is lower (up to 30%^2^) and an identifiable ‘cause’ is less likely to be found^3^. Many such fetuses fail to reach their growth potential but their birth weight is normal by population standards (i.e. fetal growth restriction (FGR)). It may be possible to identify some of these cases using ultrasound^4^. However, this is hindered by the finite availability and limited accuracy of ultrasound. A further problem is poor specificity for adverse outcomes^4^, which increases the likelihood of overintervention.

The prevention of term stillbirth nevertheless relies heavily on ultrasound, in addition to intervention in the presence of certain risk factors, such as gestational age > 41–42 weeks or prolonged prelabor rupture of membranes. With the increasing use of ultrasound in this context, complex protocols have been recommended to detect FGR, relying on estimated fetal size, Doppler indices and growth velocity, as assessed in a crude manner using centile changes^5^, ^6^. As these variables are poorly predicted by clinical and demographic risk factors^7^, universal screening remains the most comprehensive method for attempting to detect FGR at term^8^.

The benefits of universal screening are unclear^9^, ^10^, possibly because the majority of adverse outcomes at term occur in pregnancies in which placental dysfunction has not been detected. A few adverse outcomes are related to different pathology, such as fetomaternal hemorrhage, but many remain unexplained^3^. To minimize these, it is necessary to identify risk factors in pregnancies at term without SGA or conventional evidence of FGR. The aim of this analysis was to determine antepartum risk factors for fetal or neonatal mortality and severe neonatal morbidity in pregnancies in which there was no antenatal ultrasound evidence of SGA or FGR by 35–37 weeks' gestation.

## METHODS

### Study design and setting

This was a retrospective population‐based cohort study, using data from Oxford University Hospitals, Oxford, UK, of all women with a singleton pregnancy and an estimated due date between 1 October 2016 and 31 December 2023. Exclusion criteria were multiple pregnancy, fetal abnormality diagnosed prenatally, birth prior to 35 + 1 weeks, universal scan not available (owing to delivery occurring ≥ 35 + 1 weeks but before the scan), missing ultrasound or outcome data and any antenatal evidence of FGR or SGA.

In this unit, in addition to the dating/nuchal translucency scan at 11–13 weeks and the anatomy scan at 18–21 weeks, all women with a singleton pregnancy are offered a routine scan between 35 + 1 and 36 + 6 weeks, irrespective of prior risk. Fetal presentation, placental location, estimated fetal weight (EFW), amniotic fluid level, crude growth velocity since 20 weeks and fetal Doppler indices, including the pulsatility index (PI) of the umbilical artery (UA) and middle cerebral artery and the cerebroplacental ratio (CPR), are assessed at this scan. Fetal anatomy is not formally reinspected. Additional scans before and after the scan at 35 + 1 to 36 + 6 weeks are performed according to risk stratification, which involves universal uterine artery (UtA) Doppler at 18–21 weeks and clinical risk factors. Ultrasound imaging is performed by experienced sonographers or doctors according to the guidelines of the International Society of Ultrasound in Obstetrics and Gynecology (ISUOG)^11^, ^12^, using quality assurance methodology as described by the INTERGROWTH‐21^st^ project^13^. Following the scan at 35 + 1 to 36 + 6 weeks, pregnancies with suspected SGA or FGR are managed according to a published protocol^9^. This includes fetuses with an EFW < 10^th^ centile^14^; those apparently normally grown but with evidence of a ≥ 40‐centile deceleration in fetal growth; and those with UA‐PI > 95^th^ centile^15^ or abnormal CPR (a simple threshold of 1.1 was chosen for clinical practice). The impact of the universal 36‐week scan on adverse outcome has been described previously^9^, ^16^.

We intended to define an easily identifiable cohort with no evidence of SGA or FGR. To align with published and easily reproducible definitions, the exclusion criteria were: (1) Delphi consensus definition of late FGR^4^, ^5^; (2) isolated EFW < 10^th^ centile^14^; and (3) EFW ≥ 10^th^ centile with CPR < 5^th^ centile or UA‐PI > 95^th^ centile^14^, ^15^. The latter was used because previous work has shown that these pregnancies are at higher risk of adverse outcome^17^. The CPR threshold for exclusion from analysis was slightly lower (< 5^th^ centile) compared with that used clinically (single cut‐off of 1.1). Abdominal circumference growth velocity (ACGV) < 10^th^ centile^18^ between the 20‐ and 36‐week scans, but where the EFW was ≥ 10^th^ centile, was not used as an exclusion criterion because it is not used commonly and is currently impractical for a sonographer to calculate.

Routinely collected hospital data were extracted from Cerner Millennium (London, UK) for maternity data, BadgerNet (Clevermed, Edinburgh, UK) for neonatal data and ViewPoint 5 (GE Healthcare, Chicago, IL, USA) for ultrasound data.

This study was approved by the Health Research Authority (IRAS project ID: 222260; REC reference: 17/SC/0374, updated July 2024), and it was reported according to the STROBE reporting standards and guidelines.

### Risk factors

Risk factors were categorized as clinical/demographic or ultrasound‐derived. Clinical/demographic factors were maternal age, body mass index (BMI), parity, socioeconomic status, ethnicity, pregnancy‐associated plasma protein‐A (PAPP‐A) multiples of the median, smoking status, hypertensive disease, and pre‐existing and gestational diabetes. Ultrasound factors were growth velocity (measured as ACGV between the 20‐ and 36‐week scans^18^), EFW centile^14^ and UtA‐PI in mid‐pregnancy (18–21 weeks).

### Outcomes

The primary outcome was stillbirth. The secondary outcomes were: (1) a composite of severe adverse outcomes (CAO), defined as at least one of stillbirth, neonatal death < 28 days, neonatal encephalopathy Grade 2 or 3 or the need for therapeutic cooling or mechanical ventilation for > 24 h at term; and (2) severe SGA, defined as birth weight < 3^rd^ centile^19^. Stillbirths were investigated using the perinatal mortality review tool (https://www.npeu.ox.ac.uk/pmrt) in a structured fashion. ‘Unexplained’ stillbirth was defined as that for which no clear cause was found; FGR was only considered an association if placental histology was abnormal.

### Statistical analysis

Odds ratios (OR) with 95% CIs for adverse perinatal outcomes were estimated using logistic regression analysis. Where there was a significant association between a risk factor and an outcome in the univariate analysis, models were adjusted for potentially confounding factors.

Demographic data were classified into clinically relevant subgroups. Missing data for covariates were excluded where the loss was < 5%, otherwise they were included as a subgroup within that variable. Summary statistics are displayed as *n* (%) for categorical data and mean ± SD for normally distributed continuous data. Statistical analysis was performed using SPSS Statistics (version 29.0.2.0; IBM Corp., Armonk, NY, USA). Statistical significance was set at *P* < 0.05.

## RESULTS

**Figure 1:**
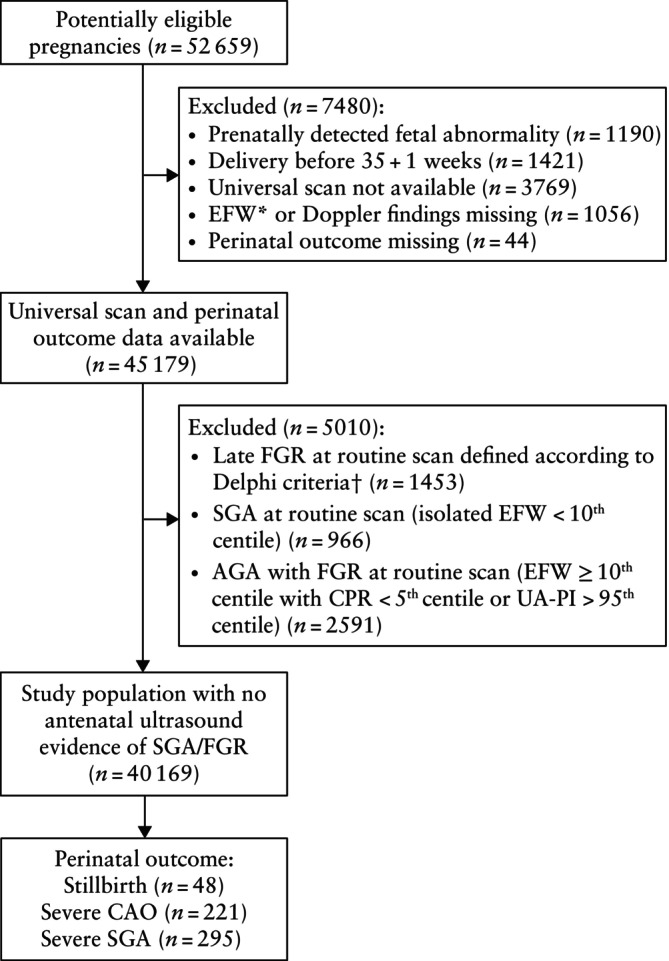
Flowchart summarizing selection of study population and perinatal outcomes of interest. *According to Hadlock formula^14^. †Estimated fetal weight (EFW) or abdominal circumference (AC) < 3^rd^ centile or presence of at least two of the following: EFW or AC < 10^th^ centile; EFW or AC crossing more than two quartiles; cerebroplacental ratio (CPR) < 5^th^ centile or umbilical artery pulsatility index (UA‐PI) > 95^th^ centile. AGA, appropriate‐for‐gestational age; CAO, composite adverse outcome; FGR, fetal growth restriction; SGA, small‐for‐gestational age.

Of 52 659 women with a singleton pregnancy during the study period, 45 179 (85.8%) were potentially eligible for inclusion, of whom 40 169 (88.9%) with no antenatal ultrasound evidence of SGA or FGR were included in the final cohort (Figure 1). There were 48 (0.1%) stillbirths, 221 (0.6%) cases of severe CAO and 295 (0.7%) cases of severe SGA at birth. The mean birth weight was 3556 g and the mean gestational age at birth was 40 + 0 weeks (Table 1). The distribution of gestational age at the late third‐trimester scan is shown in Figure S1 and the interval between the scan and birth is shown in Figure S2.

**Table 1:** Demographic and clinical characteristics of 40 169 pregnancies with no ultrasound evidence of small‐for‐gestational age or fetal growth restriction

Characteristic	Value
Maternal age (years)	31.4 ± 5.28
Maternal BMI (kg/m^2^)*	25.8 ± 5.55
Maternal BMI category	
Underweight (< 18.5 kg/m^2^)	1032/39 882 (2.6)
Normal weight (18.5–24.9 kg/m^2^)	20 363/39 882 (51.1)
Overweight (25.0–29.9 kg/m^2^)	10 910/39 882 (27.4)
Class‐1 obesity (30.0–34.9 kg/m^2^)	4715/39 882 (11.8)
Class‐2–3 obesity (≥ 35.0 kg/m^2^)	2862/39 882 (7.2)
Smoker during pregnancy	2898 (7.2)
Ethnicity	
White	33 316 (82.9)
Black	1046 (2.6)
South Asian	2079 (5.2)
Asian	1543 (3.8)
Mixed	907 (2.3)
Other or unknown	1278 (3.2)
Nulliparous	18 037 (44.9)
Pre‐existing diabetes	224 (0.6)
Gestational diabetes	2045 (5.1)
Chronic hypertension	428 (1.1)
PIH	1092 (2.7)
Pre‐eclampsia	1430 (3.6)
PAPP‐A (MoM)†	1.16 ± 0.65
PAPP‐A < 0.41 MoM	1458/28 729 (5.1)
IMD decile	
1–2 (most deprived)	1909/38 990 (4.9)
3–4	3530/38 990 (9.1)
5–6	6348/38 990 (16.3)
7–8	10 820/38 990 (27.8)
9–10 (least deprived)	16 383/38 990 (42.0)
GA at delivery (days)	280 ± 9
Birth weight (g)	3556 ± 457
Birth‐weight centile^19^	57 ± 27

Data are given as mean ± SD, *n/N* (%) or *n* (%).

* Data missing for 287 patients.

† Data missing for 11 440 patients (declined or missed screening). BMI, body mass index; GA, gestational age; IMD, index of multiple deprivation; MoM, multiples of the median; PAPP‐A, pregnancy‐associated plasma protein‐A; PIH, pregnancy‐induced hypertension.

Table 2 shows the key causes of, and risk factors associated with, the 48 cases of stillbirth. Twenty‐three (47.9%) stillbirths were considered unexplained. A relative relationship map illustrating the multivariable associations with stillbirth is provided in Figure S3. Of the seven (14.6%) intrapartum deaths, three occurred outside of the hospital. Among stillbirths, five (10.4%) cases had a birth weight < 10^th^ centile (adjusted odds ratio (aOR), 2.70 (95% CI, 1.06–6.92)) and none had a birth weight < 3^rd^ centile. Fifteen (6.8%) pregnancies with severe CAO had a birth weight < 10^th^ centile (aOR, 1.74 (95% CI, 1.02–2.97)), while 46 (20.8%) were large‐for‐gestational age (LGA) (birth weight > 90^th^ centile) (aOR, 1.68 (95% CI, 1.19–2.38)). Risk factors at birth for stillbirth, severe CAO and severe SGA are summarized in Table S1.

**Table 2:** Causes of and risk factors associated with stillbirth in 48 pregnancies with no ultrasound evidence of small‐for‐gestational age or fetal growth restriction (FGR)

Characteristic	*n* (%)
Nulliparous	28 (58.3)
Maternal BMI ≥ 35.0 kg/m^2^	6 (12.5)
Pre‐existing diabetes	1 (2.1)
Pre‐eclampsia	7 (14.6)
AGA with possible FGR	3 (6.3)
BW < 10^th^ centile	5 (10.4)
BW < 3^rd^ centile	0 (0)
UtA‐PI > 95^th^ centile at 18–21 weeks	4 (8.3)
Vasa previa	1 (2.1)
Event not predictable on ultrasound	13 (27.1)
Placental abruption with no evidence of hypertension	5 (10.4)
Cord accident	1 (2.1)
Chorioamnionitis	6 (12.5)
Fetomaternal hemorrhage	1 (2.1)
Unexplained stillbirth	23 (47.9)
Without risk factors	17 (35.4)
With at least one risk factor	6 (12.5)
GA at delivery > 41 weeks	9 (18.8)
Intrapartum death	7 (14.6)

Characteristics are not mutually exclusive. AGA, appropriate‐for‐gestational age; BMI, body mass index; BW, birth weight; GA, gestational age; UtA‐PI, mean uterine artery pulsatility index.

### Antenatal prediction of stillbirth

Potential antenatal clinical/demographic and ultrasonographic risk factors for stillbirth are shown in Table 3. Pre‐eclampsia was the only risk factor that was associated significantly with term stillbirth (OR, 4.46 (95% CI, 1.99–9.96)). Based on the prevalence of pre‐eclampsia of 14.6% among stillbirth cases, the population‐attributable fraction of pre‐eclampsia was 9.6%; the absolute risk of stillbirth with pre‐eclampsia was 0.5%. None of the ultrasonographic variables, including those assessing growth velocity, were a significant risk factor for stillbirth.

**Table 3:** Univariate logistic regression analysis of maternal and ultrasound risk factors for stillbirth

Characteristic	Total (*n*) (*n* = 40 169)	Stillbirth (*n* = 48)*	No stillbirth (*n* = 40 121)*	OR (95% CI)
Maternal age				
20–40 years	38 162	44 (0.1)	38 118 (99.9)	1.00 (ref)
> 40 years	1356	3 (0.2)	1353 (99.8)	1.92 (0.60–6.19)
< 20 years	651	1 (0.2)	650 (99.8)	1.33 (0.18–9.69)
Ethnicity				
White	33 316	38 (0.1)	33 278 (99.9)	1.00 (ref)
Black	1046	0 (0)	1046 (100)	—
South Asian	2079	4 (0.2)	2075 (99.8)	1.69 (0.60–4.73)
Asian	1543	2 (0.1)	1541 (99.9)	1.14 (0.27–4.71)
Mixed	907	1 (0.1)	906 (99.9)	0.97 (0.13–7.05)
Other or unknown	1278	3 (0.2)	1275 (99.8)	2.06 (0.63–6.68)
IMD decile†				
3–10	37 081	43 (0.1)	37 038 (99.9)	1.00 (ref)
1–2 (most deprived)	1909	2 (0.1)	1907 (99.9)	0.90 (0.22–3.73)
Maternal BMI ≥ 35.0 kg/m^2^	2862	6 (0.2)	2856 (99.8)	1.85 (0.79–4.35)
Smoker during pregnancy	2898	4 (0.1)	2894 (99.9)	1.17 (0.42–3.26)
Nulliparous	18 037	28 (0.2)	18 009 (99.8)	1.72 (0.97–3.05)
Glucose metabolism				
Non‐impaired	37 900	44 (0.1)	37 856 (99.9)	1.00 (ref)
Pre‐existing diabetes	224	1 (0.4)	223 (99.6)	3.86 (0.53–28.12)
Gestational diabetes	2045	3 (0.1)	2042 (99.9)	1.26 (0.39–4.07)
PAPP‐A level				
≥ 0.41 MoM	27 275	29 (0.1)	27 246 (99.9)	1.00 (ref)
< 0.41 MoM	1458	2 (0.1)	1456 (99.9)	1.29 (0.31–5.41)
Unknown	11 436	17 (0.1)	11 419 (99.9)	1.40 (0.77–2.54)
Hypertensive disorder				
None	37 219	41 (0.1)	37 178 (99.9)	1.00 (ref)
Chronic hypertension	428	0 (0)	428 (100)	—
PIH	1092	0 (0)	1092 (100)	—
Pre‐eclampsia	1430	7 (0.5)	1423 (99.5)	4.46 (1.99–9.96)
EFW‡				
20^th^ to < 90^th^ centile	34 893	40 (0.1)	34 853 (99.9)	1.00 (ref)
10^th^ to < 20^th^ centile	3242	5 (0.2)	3237 (99.8)	1.35 (0.53–3.41)
≥ 90^th^ centile	2034	3 (0.1)	2031 (99.9)	1.29 (0.40–4.16)
ACGV at 20–36 weeks§				
10–90^th^ centile	32 273	36 (0.1)	32 237 (99.9)	1.00 (ref)
> 90^th^ centile	3115	5 (0.2)	3110 (99.8)	1.40 (0.55–3.57)
< 10^th^ centile	2925	5 (0.2)	2920 (99.8)	1.49 (0.59–3.80)
UtA‐PI > 95^th^ centile at 18–21 weeks	1646	4 (0.2)	1642 (99.8)	2.07 (0.74–5.78)

* Data are given as *n* (%), where percentages are calculated row‐wise.

† Data missing for 1179 patients.

‡ According to Hadlock formula^14^.

§ Data missing for 1856 patients. ACGV, abdominal circumference growth velocity; BMI, body mass index; EFW, estimated fetal weight; IMD, index of multiple deprivation; MoM, multiples of the median; OR, odds ratio; PAPP‐A, pregnancy‐associated plasma protein‐A; PIH, pregnancy‐induced hypertension; ref, reference; UtA‐PI, mean uterine artery pulsatility index.

### Prediction of severe composite adverse outcome

Potential clinical and ultrasonographic risk factors for severe CAO are shown in Table 4. In the univariate analysis, high BMI, nulliparity, pre‐existing and gestational diabetes, pre‐eclampsia and EFW ≥ 90^th^ centile were associated significantly with severe CAO but, after adjustment for confounding factors, only pre‐existing diabetes (aOR, 2.82 (95% CI, 1.02–7.85)) and nulliparity (aOR, 1.56 (95% CI, 1.19–2.04)) remained significant.

**Table 4:** Uni‐ and multivariate regression analysis of maternal and ultrasound risk factors for severe composite adverse outcome (CAO)

Characteristic	Total (*n*) (*n* = 40 169)	Severe CAO (*n* = 221)*	No severe CAO (*n* = 39 948)*	OR (95% CI)	aOR (95% CI)
Maternal age					
20–40 years	38 162	208 (0.5)	37 954 (99.5)	1.00 (ref)	—
> 40 years	1356	9 (0.7)	1347 (99.3)	1.22 (0.62–2.38)	—
< 20 years	651	4 (0.6)	647 (99.4)	1.13 (0.42–3.04)	—
Ethnicity					
White	33 316	178 (0.5)	33 138 (99.5)	1.00 (ref)	—
Black	1046	8 (0.8)	1038 (99.2)	1.43 (0.70–2.92)	—
South Asian	2079	17 (0.8)	2062 (99.2)	1.53 (0.93–2.53)	—
Asian	1543	8 (0.8)	1535 (99.2)	0.97 (0.48–1.97)	—
Mixed	907	6 (0.7)	901 (99.3)	1.24 (0.55–2.80)	—
Other or unknown	1278	4 (0.3)	1274 (99.7)	0.58 (0.22–1.58)	—
IMD decile†					
3–10	37 081	206 (0.6)	36 875 (99.4)	1.00 (ref)	—
1–2 (most deprived)	1909	8 (0.4)	1901 (99.6)	0.75 (0.37–1.53)	—
Maternal BMI ≥ 35.0 kg/m^2^	2862	24 (0.8)	2838 (99.2)	1.59 (1.04–2.44)	1.42 (0.91–2.27)¶
Smoker during pregnancy	2898	15 (0.5)	2883 (99.5)	0.94 (0.55–1.58)	—
Nulliparous	18 037	123 (0.7)	17 914 (99.3)	1.54 (1.18–2.01)	1.56 (1.19–2.04)¶
Glucose metabolism					
Non‐impaired	37 900	199 (0.5)	37 701 (99.5)	1.00 (ref)	—
Pre‐existing diabetes	224	4 (1.8)	220 (98.2)	3.44 (1.27–9.35)	2.82 (1.02–7.85)**
Gestational diabetes	2045	18 (0.9)	2027 (99.1)	1.68 (1.03–2.73)	1.53 (0.93–2.51)††
PAPP‐A level					
≥ 0.41 MoM	27 275	145 (0.5)	27 130 (99.5)	1.00 (ref)	—
< 0.41 MoM	1458	11 (0.8)	1447 (99.2)	1.42 (0.77–2.63)	—
Unknown	11 436	65 (0.6)	11 371 (99.4)	1.07 (0.80–1.43)	—
Hypertensive disorder					
None	37 219	195 (0.5)	37 024 (99.5)	1.00 (ref)	—
Chronic hypertension	428	2 (0.5)	426 (99.5)	0.89 (0.22–3.60)	—
PIH	1092	10 (0.9)	1082 (99.1)	1.75 (0.93–3.32)	—
Pre‐eclampsia	1430	14 (1.0)	1416 (99.0)	1.88 (1.09–3.24)	1.65 (0.95–2.88)‡‡
EFW‡					
20^th^ to < 90^th^ centile	34 893	185 (0.5)	34 708 (99.5)	1.00 (ref)	—
10^th^ to < 20^th^ centile	3242	18 (0.6)	3224 (99.4)	1.05 (0.64–1.70)	—
≥ 90^th^ centile	2034	18 (0.9)	2016 (99.1)	1.67 (1.03–2.72)	1.52 (0.90–2.56)§§
ACGV at 20–36 weeks§					
10–90^th^ centile	32 273	172 (0.5)	32 101 (99.5)	1.00 (ref)	—
> 90^th^ centile	3115	22 (0.7)	3093 (99.3)	1.33 (0.85–2.07)	—
< 10^th^ centile	2925	16 (0.5)	2909 (99.5)	1.03 (0.61–1.72)	—
UtA‐PI > 95^th^ centile at 18–21 weeks	1646	9 (0.5)	1637 (99.5)	0.99 (0.51–1.94)	—

CAO was defined as at least one of: stillbirth; neonatal death < 28 days; neonatal encephalopathy Grade 2 or 3; or need for therapeutic cooling or mechanical ventilation for > 24 h at term.

* Data are given as *n* (%), where percentages are calculated row‐wise.

† Data missing for 1179 patients.

‡ According to Hadlock formula^14^.

§ Data missing for 1856 patients.

¶ Adjusted for body mass index (BMI) ≥ 35.0 kg/m^2^, estimated fetal weight (EFW) ≥ 90^th^ centile, pre‐eclampsia, pre‐existing and gestational diabetes and nulliparity.

** Adjusted for BMI ≥ 35.0 kg/m^2^, EFW ≥ 90^th^ centile, pre‐eclampsia, pre‐existing diabetes and nulliparity.

†† Adjusted for BMI ≥ 35.0 kg/m^2^, EFW ≥ 90^th^ centile, pre‐eclampsia, gestational diabetes and nulliparity.

‡‡ Adjusted for BMI ≥ 35.0 kg/m^2^, pre‐eclampsia, pre‐existing and gestational diabetes, and nulliparity.

§§ Adjusted for BMI ≥ 35.0 kg/m^2^, EFW ≥ 90^th^ centile, pre‐existing and gestational diabetes, and nulliparity. ACGV, abdominal circumference growth velocity; aOR, adjusted odds ratio; IMD, index of multiple deprivation; MoM, multiples of the median; OR, odds ratio; PAPP‐A, pregnancy‐associated plasma protein‐A; PIH, pregnancy‐induced hypertension; ref, reference; UtA‐PI, mean uterine artery pulsatility index.

### Severe SGA

Demographic and clinical risk factors for severe SGA were Black and South Asian ethnicity, smoking during pregnancy, nulliparity and low levels of PAPP‐A, which were statistically significant in both the univariate and adjusted analyses (Table 5). Low maternal age and pre‐eclampsia were significant risk factors in the univariate analysis, but not after adjustment for confounding factors. Significant ultrasound risk factors were raised UtA‐PI in the mid‐trimester, EFW 10^th^ to < 20^th^ centile and ACGV < 10^th^ centile.

**Table 5:** Uni‐ and multivariate regression analysis of maternal and ultrasound risk factors for severe small‐for‐gestational age (SGA) (birth weight < 3^rd^ centile^19^)

Characteristic	Total (*n*) (*n* = 40 169)	Severe SGA (*n* = 295)*	No severe SGA (*n* = 39 874)*	OR (95% CI)	aOR (95% CI)†
Maternal age					
20–40 years	38 162	278 (0.7)	37 884 (99.3)	1.00 (ref)	—
> 40 years	1356	7 (0.5)	1349 (99.5)	0.71 (0.33–1.50)	—
< 20 years	651	10 (1.5)	641 (98.5)	2.13 (1.13– 4.01)	1.33 (0.56–3.14)
Ethnicity					
White	33 316	205 (0.6)	33 111 (99.4)	1.00 (ref)	—
Black	1046	14 (1.3)	1032 (98.7)	2.19 (1.27–3.78)	3.11 (1.59–6.10)
South Asian	2079	40 (1.9)	2039 (98.1)	3.17 (2.25–4.46)	2.99 (1.92–4.66)
Asian	1543	14 (0.9)	1529 (99.1)	1.48 (0.86–2.55)	—
Mixed	907	9 (1.0)	898 (99.0)	1.62 (0.83–3.17)	—
Other or unknown	1278	13 (1.0)	1265 (99.0)	1.66 (0.94–2.92)	—
IMD decile‡					
3–10	37 081	265 (0.7)	36 816 (99.3)	1.00 (ref)	—
1–2 (most deprived)	1909	21 (1.1)	1888 (98.9)	1.55 (0.99–2.42)	—
Maternal BMI ≥ 35.0 kg/m^2^	2862	24 (0.8)	2838 (99.2)	1.15 (0.76–1.75)	—
Smoker during pregnancy	2898	54 (1.9)	2844 (98.1)	2.92 (2.17–3.93)	3.04 (2.07–4.45)
Nulliparous	18 037	187 (1.0)	17 850 (99.0)	2.14 (1.68–2.71)	2.29 (1.70–3.08)
Glucose metabolism					
Non‐impaired	37 900	285 (0.8)	37 615 (99.2)	1.00 (ref)	—
Pre‐existing diabetes	224	0 (0)	224 (100)	—	—
Gestational diabetes	2045	10 (0.5)	2035 (99.5)	0.65 (0.34–1.22)	—
PAPP‐A level					
≥ 0.41 MoM	27 275	186 (0.7)	27 089 (99.3)	1.00 (ref)	—
< 0.41 MoM	1458	22 (1.5)	1436 (98.5)	2.23 (1.43–3.48)	1.86 (1.17–2.95)
Unknown	11 436	87 (0.8)	11 349 (99.2)	1.12 (0.86–1.44)	—
Hypertensive disorder					
None	37 219	265 (0.7)	36 954 (99.3)	1.00 (ref)	—
Chronic hypertension	428	2 (0.5)	426 (99.5)	0.65 (0.16–2.64)	—
PIH	1092	10 (0.9)	1082 (99.1)	1.29 (0.68–2.43)	—
Pre‐eclampsia	1430	18 (1.3)	1412 (98.7)	1.78 (1.10–2.87)	1.43 (0.80–2.58)
EFW§					
20^th^ to < 90^th^ centile	34 893	149 (0.4)	34 744 (99.6)	1.00 (ref)	—
10^th^ to < 20^th^ centile	3242	146 (4.5)	3096 (95.5)	10.97 (8.73–13.86)	9.45 (7.11–12.54)
≥ 90^th^ centile	2034	0 (0)	2034 (100)	—	—
ACGV at 20–36 weeks¶					
10–90^th^ centile	32 273	228 (0.7)	32 045 (99.3)	1.00 (ref)	—
> 90^th^ centile	3115	8 (0.3)	3107 (99.7)	0.36 (0.18–0.73)	—
< 10^th^ centile	2925	49 (1.7)	2876 (98.3)	2.39 (1.75–3.27)	1.57 (1.06–2.32)
UtA‐PI > 95^th^ centile at 18–21 weeks	1646	36 (2.2)	1610 (97.8)	3.30 (2.30–4.65)	2.76 (1.82–4.19)

* Data are given as *n* (%), where percentages are calculated row‐wise.

† Adjusted for maternal age < 20 years, black or South Asian ethnicity, smoker during pregnancy, nulliparity, pregnancy‐associated plasma protein‐A (PAPP‐A) < 0.41 multiples of the median (MoM), pre‐eclampsia, estimated fetal weight (EFW) 10^th^ to < 20^th^ centile, abdominal circumference growth velocity (ACGV) < 10^th^ centile and mean uterine artery pulsatility index (UtA‐PI) > 95^th^ centile at 18–21 weeks.

‡ Data missing for 1179 patients.

§ According to Hadlock formula^14^.

¶ Data missing for 1856 patients. aOR, adjusted odds ratio; BMI, body mass index; IMD, index of multiple deprivation; OR, odds ratio; PIH, pregnancy‐induced hypertension; ref, reference.

## DISCUSSION

This analysis identified the few clinically important risk factors for adverse outcomes at term in the low‐risk majority subgroup of a population screened comprehensively for FGR and SGA using established criteria at a late third‐trimester ultrasound scan. Therefore, our findings are representative of a sophisticated but real‐world setting.

### Fetal size and growth

Although present among cases with adverse outcome, low birth weight made a smaller contribution to mortality and morbidity than what is usually quoted. This is presumably attributable to the earlier identification of FGR and SGA. Failure to detect the remaining cases could be due to a reduction in growth after the 36‐week scan or well‐documented scan inaccuracy. Fetal biometry has poor sensitivity for SGA; while this is improved by using markers of FGR, the sensitivity remains, at best, little more than 50%^9^, ^20^. This was shown in the remaining proportion of infants who were delivered with birth weight below the 3^rd^ centile (*n* = 295 (0.7%)).

There are other risk factors for severe SGA, most of which have been established in the literature. The finding that EFW 10^th^ to < 20^th^ centile constitutes a risk for severe SGA is not surprising. Nevertheless, the limited role of SGA in mortality and severe morbidity undermines the potential role for further SGA detection after a ‘normal’ 36‐week scan. Furthermore, it has been postulated that the role of SGA in stillbirth has been exaggerated because of fetal weight loss between demise and delivery^21^.

Therefore, the finding that just under 90% of stillborn babies were not SGA at delivery (i.e. birth weight was ≥ 10^th^ centile) is important. Only pre‐eclampsia was a significant risk factor for stillbirth, and only nulliparity and pre‐existing diabetes were risk factors for severe CAO. It is possible that some cases had growth deceleration, although the lack of association between severe CAO and low ACGV, a possible marker for adverse outcome^18^, does not support this. Nevertheless, it has been reported previously that slowed growth in the third trimester is associated with stillbirth in non‐SGA fetuses^22^, and we do not have data on late growth alteration.

Other ultrasound markers that may relate to growth aberration, including mid‐trimester UtA‐PI, were also found not to be clinically important risk factors. This could be because of an intervention paradox^23^, as raised UtA‐PI has been associated with term stillbirth^24^, and the unit induction guidelines take account of this^9^. Indeed, the association of abnormal UtA‐PI with severe SGA at birth suggests persisting risk. We do not have data on other fetal Doppler parameters, particularly CPR, which is known to be associated with worse outcome even in appropriate‐for‐gestational‐age (AGA) infants^17^, because abnormal values at the 36‐week scan were considered an exclusion criterion. Given that CPR is more predictive of adverse outcome when measured closer to the birth^25^, ^26^, it is possible that later and more frequent assessment may have predictive value^25^.

The subtleties of FGR and the low‐level persistence of SGA among cases of stillbirth and severe CAO suggest that, in addition to improvements in scan accuracy, later ultrasound assessment, particularly with reference to growth trajectory, could have a limited role in the prediction of adverse perinatal outcome. However, it is unclear which pregnancies would benefit from this, and the false‐positive rate would pose the same issues as those now associated with the detection of SGA^3^.

### Other risk factors

Despite their limited role, the risk factors identified herein require attention if perinatal outcomes are to be improved. Pre‐eclampsia accounted for nearly 10% of stillbirths. It was less clearly associated with severe CAO, despite its known association with encephalopathy^27^. The International Society for the Study of Hypertension in Pregnancy criteria for pre‐eclampsia imply that, by definition, a pregnancy with new hypertension that ends in stillbirth is pre‐eclamptic^28^. In our dataset, three pregnancies with apparent pregnancy‐induced hypertension were pre‐eclamptic solely according to this definition. This, and the unclear timing of manifestation, limit the usefulness of pre‐eclampsia as a risk factor for stillbirth, although this might be improved using biomarker testing^29^. Our findings nevertheless emphasize the need to expedite birth urgently if pre‐eclampsia is diagnosed after 37 weeks, irrespective of ultrasound findings.

Pre‐existing diabetes was found to be a significant risk factor for severe CAO. Guidelines for diabetes advocate birth before 39 weeks, but mortality and morbidity were present despite adherence to this^30^. Nulliparity was the only other independent risk factor for severe CAO but, owing to the high frequency of this characteristic, it could only be used in practice as part of a model.

The possible increased risk of severe CAO associated with LGA at birth is probably due to an intrapartum role. This may be less subject to intervention paradox because local practice reflects the prevailing evidence that expediting the birth of a large fetus has limited benefit^31^. As with SGA fetuses, scan accuracy is a problem in this context^32^. Although previous work suggests that it is the increased growth velocity rather than estimated size that is important^33^, at least for less severe adverse outcomes, our data do not confirm this. This all means that the capacity for improvement is small. Further investigation into which LGA fetuses are at highest risk is required.

### Strengths and limitations

Despite its size, this analysis of clinically important risk factors for adverse outcomes is limited by their low prevalence and the retrospective study design. Owing to the lack of blinding, our study was subject to intervention paradox, which may have attenuated associations with factors thought to confer risk. We chose to include all causes of stillbirth, rather than solely ‘placenta‐mediated’ stillbirth, because this imprecise categorization prevents analysis of the outcome that matters most, i.e. any stillbirth. The fact that our definition of unexplained stillbirth meant that this accounted for nearly half of term stillbirths does not detract from the analysis. Moreover, the rate of missing information was low, except in the case of PAPP‐A, owing to declined aneuploidy screening. Despite standardized training and audit, scan accuracy is imperfect. Although this does reflect real‐world practice, it is an area requiring improvement. Additionally, our definitions of FGR and SGA, which led to the exclusion of 11% of eligible pregnancies, differed slightly from those used for management. In clinical practice, we considered a CPR threshold of 1.1 for referral for further assessment in AGA fetuses, whereas CPR < 5^th^ centile was used in our analysis^15^. Furthermore, growth deceleration was defined as > 40 centiles (rather than > 50 centiles as recommended by ISUOG) and the ACGV was not calculated. Given that centile changes are now recognized to have poor clinical utility owing to the mathematical superiority of *Z‐*scores over centiles, this means that some fetuses with growth deceleration, but not EFW < 10^th^ centile, were included. While these cases slightly mitigate the effect of intervention paradox, they allow the cohort to be easily replicated and represent common clinical practice: ACGV and other *Z*‐score‐based definitions of growth velocity are not easily performed in a practical setting but can be assessed as a risk factor. Additionally, owing to the low rate of non‐White ethnicity in our dataset (13.9%), we were unable to determine the importance of ethnicity, and this limits the translation of our findings to a more ethnically diverse population. Finally, our findings also apply only to a similar setting in which a comprehensive policy of FGR detection is attempted, but that is the point: attempts to identify FGR are already recommended widely, albeit in differing ways.

### Conclusions

The absence of risk factors for perinatal morbidity and mortality in our population of pregnancies without antenatal evidence of SGA or FGR is striking. This finding should only be considered relevant in a population that has been ‘screened’ for FGR, but it means that the prediction of stillbirth and severe morbidity in apparently non‐FGR fetuses is poor. This is because of our limited understanding of the multiple and complex etiologies underpinning mortality and morbidity. Comparative biometry and perhaps more frequent CPR assessment might identify more at‐risk fetuses, but with associated false positives and unnecessary interventions. Better identification of pre‐eclampsia and rare conditions such as vasa previa, as well as improved management of diabetes, may help too. Nevertheless, the lack of risk factors suggests the need for more data and modeling using multiple risk factors. Until this is done, it is difficult not to infer that routine early‐term expediting of birth, a policy that is unpopular, requires more staffing, is often associated with a worse maternal experience^34^ and may be detrimental to long‐term health^35^, but clearly reduces perinatal mortality^36^, is the principal available method for further reducing adverse outcomes at term.

## Supporting information


**Figure S1** Distribution of gestational age at scan (in days) in study population.

**Figure S2** Distribution of time interval (in days) between scan and delivery/diagnosis of stillbirth in study population.

**Figure S3** Relationship between causes of and risk factors associated with stillbirth.

**Table S1** Risk factors at birth for stillbirth, severe composite adverse outcome and severe small‐for‐gestational age

## Data Availability

The data that support the findings of this study are available from the corresponding author upon reasonable request.
