# Supplementary figures and images for: Risk factors for mortality and severe morbidity in fetuses with normal late third‐trimester scan: population‐based cohort study
Source: Ultrasound Obstet Gynecol. 2025 Jun 16;66(1):56–64. doi: 10.1002/uog.29256 (PMC12209687; doi:10.1002/uog.29256)

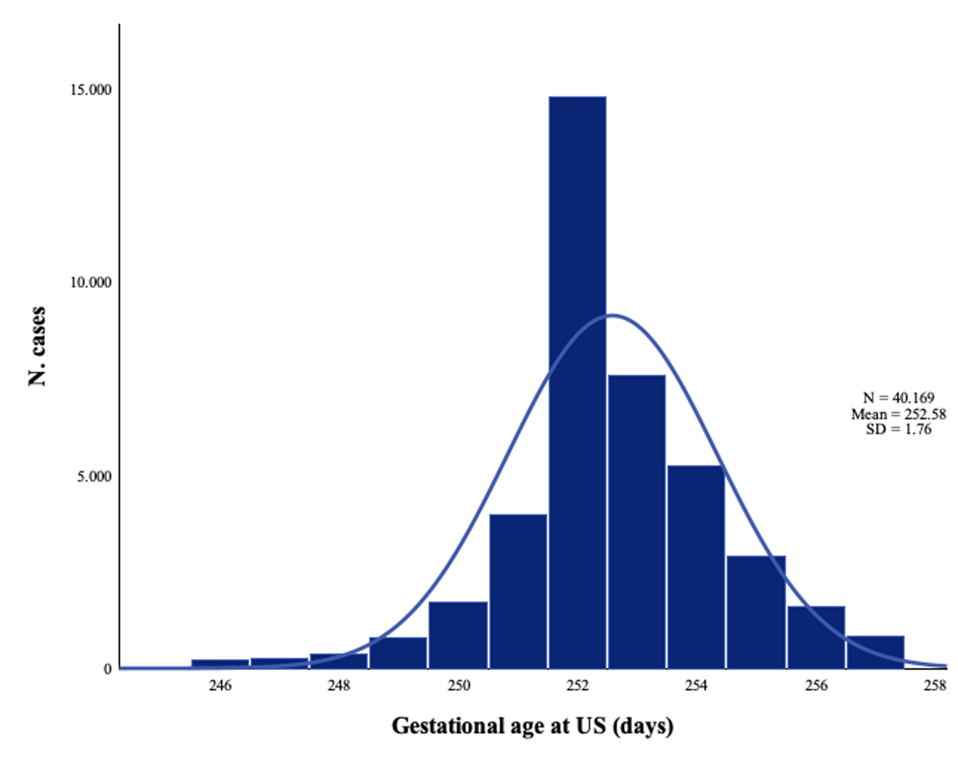

Supplement: Supplementary file 1 — Figure S1 Distribution of gestational age at scan (in days) in study population. [file UOG-66-56-s004.png]

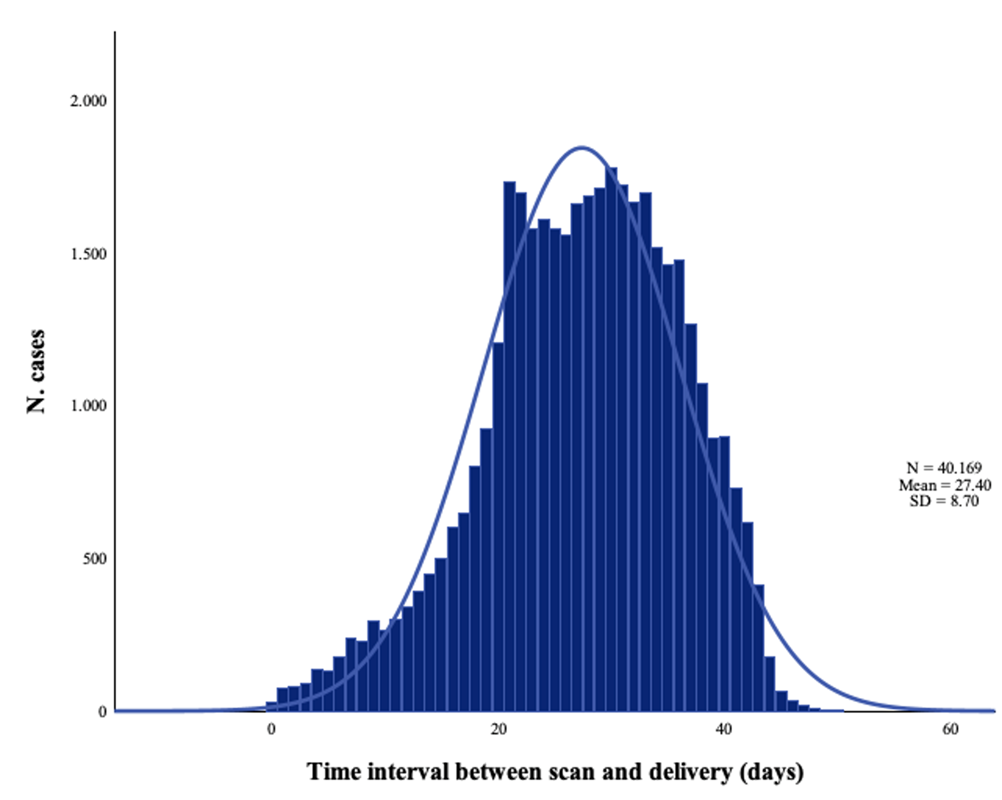

Supplement: Supplementary file 2 — Figure S2 Distribution of time interval (in days) between scan and delivery/diagnosis of stillbirth in study population. [file UOG-66-56-s003.png]

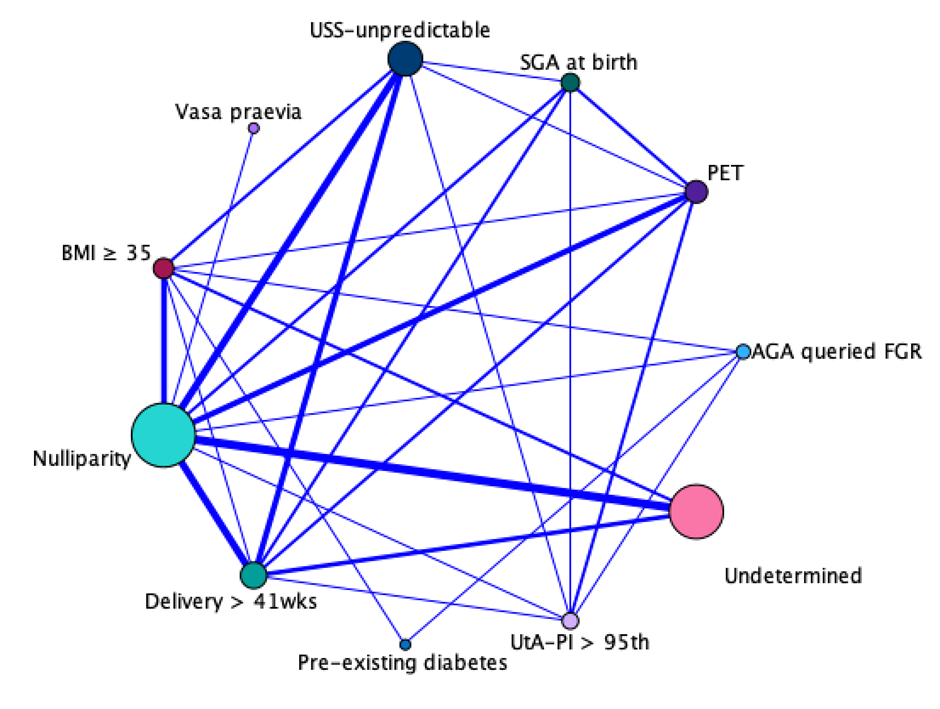

Supplement: Supplementary file 3 — Figure S3 Relationship between causes of and risk factors associated with stillbirth. [file UOG-66-56-s002.png]
