# Supplementary material for: Risk factors for mortality and severe morbidity in fetuses with normal late third‐trimester scan: population‐based cohort study
Source: Ultrasound Obstet Gynecol. 2025 Jun 16;66(1):56–64. doi: 10.1002/uog.29256 (PMC12209687; doi:10.1002/uog.29256)
Supplement: Supplementary file 4 — Table S1 Risk factors at birth for stillbirth, severe composite adverse outcome and severe small‐for‐gestational age [file UOG-66-56-s001.docx]

**Table S1.** Risk factors at birth for stillbirth, severe composite adverse outcome and severe small-for-gestational age

| ***Characteristics*** |  |  | | **ORs (CI 95%)** | | **aORs (95% CI)** | | |
| --- | --- | --- | --- | --- | --- | --- | --- | --- |
|  |  | **Stillbirth** | |  |  |  |  |  |
|  | **Total** | **SB**  **(N=48)** | **No SB**  **(N=40121)** | **OR** | **CI 95%** | **aOR** | **CI 95%** | |
| **Birth > 41 wks** | 8311 | 9 (0.1%) | 8302 (99.9%) | 0.88 | (0.43 – 1.83) | - | - | |
| **10^th^ ≥ BW**  ≤ **90^th^ centile** | 33122 | 35 (0.1%) | 33087 (99.9%) | 1.00 (ref) | - | - | - | |
| **BW < 10^th^ centile** | 1657 | 5 (0.3%) | 1652 (99.7%) | **2.86** | **(1.12 – 7.31)** | **2.70^1^** | **(1.06 – 6.92)** | |
| **BW > 90^th^ centile** | 5390 | 8 (0.1%) | 5382 (99.9%) | 1.40 | (0.65 – 3.03) | - | - | |
|  |  | **Severe CAO** | |  |  |  |  |  |
|  | **Total** | **Severe CAO**  **(N=221)** | **No severe CAO (N=39948)** | **OR** | **CI 95%** | **aOR** | **CI 95%** | |
| **Birth > 41 wks** | 8311 | 59 (0.7%) | 8252 (99.3%) | **1.40** | **(1.04 – 1.89)** | 1.42^2^ | (1.05 – 1.94) | |
| **10^th^ ≥ BW**  ≤ **90^th^ centile** | 33122 | 160 (0.5%) | 32962 (99.5%) | 1.00 (ref) | **-** | - | - | |
| **BW < 10^th^ centile** | 1657 | 15 (0.9%) | 1642 (99.1%) | **1.88** | **(1.01 – 3.20)** | **1.74^2^** | **(1.02 – 2.97)** | |
| **BW > 90^th^ centile** | 5390 | 46 (0.9%) | 5345 (99.1%) | **1.77** | **(1.28 – 2.46)** | **1.68^2^** | **(1.19 – 2.38)** | |
|  |  | **Severe SGA** | |  |  |  |  |  |
|  | **Total** | **Severe SGA (N=295)** | **No severe SGA (N=39874)** | **OR** | **CI 95%** | **aOR** | **CI 95%** | |
| **Birth > 41 wks** | 8311 | 72 (0.9%) | 8239 (99.1%) | 1.24 | (0.95 – 1.62) | **-** | **-** | |

Data are provided as N(%), odds ratio (OR) and adjusted OR with 95% confidence intervals (CI) according to the univariate and multivariate logistic regression; BW, birthweight; CAO, composite adverse outcome; SB, stillbirth; SGA, small-for-gestational ag. Covariates used for adjustment in the multivariate logistic regression: 1) PET; 2) BMI ≥ 35, nulliparity, impaired glucose metabolism, PET, EFW > 90^th^ and birthweight
